# Notes on Practical Haematology

**Published:** 1910-01-08

**Authors:** 


### Pathology.

### NOTES ON PRACTICAL HÆMATOLOGY.

### APPARATUS FOR BLOOD EXAMINATION.

For the examination of blood no very extensive apparatus is required beyond, of course, a good microscope. A hæmocytometer (Thomas Zeiss) costs only 30s.; there are plenty of good hæmoglobinometers on the market, and cover glasses, slides, and stains are about the only other outlay. The microscope should have three objectives, $\frac{2}{3}$-inch, $\frac{1}{6}$-inch, and $\frac{1}{12}$-inch), two eye-pieces, an iris diaphragm, and Abbè condenser, and a mechanical stage: the extra expense of the latter is easily recompensed by its great convenience. The complete instrument will cost £18 to £20: most of the leading makers now producing such an article at or about this price. All glass has a tendency to deteriorate in the tropics, so special attention must be paid to objectives, cover-glasses and slides in such climates. Cover-glasses must not be taken out dry, they can be packed either in clove oil or alcohol; care should be taken that the latter has no chance of evaporating. The second quality (green glass) of slide keeps well, so it should be employed in place of the first quality, which goes rapidly. Objectives must be wiped carefully with a soft silk handkerchief after use, and then they should be kept till required again in a desiccator to which calcium chloride has been added. This, of course, applies equally to photographic and telescopic lenses, which are also liable to become frosted. Once this change takes place, no amount of rubbing or cleansing the glass will remove it; it has to be sent to the maker and reground, and this, of course, takes a long time. Slides and cover-glasses, wherever they are to be used, must be thoroughly clean; neglect of this leads to slovenly work, and artefarcts become so numerous that they are often the cause of mistakes. Van Ermengem's method for cleaning cover-glasses may be employed. He recommends a solution consisting of the following reagents:—

| | | |
|---|---|---|
| I. | Sulphuric acid ($H_2S.O_4$) concentrated | 6 parts. |
| | Potassium bichromate ... ... | 6 parts. |
| | Water ... ... ... ... | 100 parts. |

Cover-glasses are placed in this mixture for twenty-four hours; after this they are washed thoroughly in water, then stored in absolute alcohol.

II. Another useful method is to boil the cover-glasses in water, to which soda has been added. Wash well afterwards, and store in methylated spirit. Slides should also be treated in this manner and then stored in spirit.

III. Another method is to place the cover-glasses or slides in pure hydrochloric acid, keep in this for twenty-four hours, then wash well; store in spirits.

Slides, and especially cover-glasses, as they come from the makers are often dirty and greasy, so it is essential for good work that they should have this removed before use; this may be accomplished by any of the above methods. The advantage of storing in alcohol is easily apparent; on taking them out the fluid quickly evaporates of its own accord or can be rubbed off cleanly with a silk handkerchief; others prefer, in the case of cover-glasses, to hold them over the bunsen and let the spirit burn off.

#### Examination of Fresh Blood.

The blood may be drawn from any part of the body, the ear and finger being the most usually selected parts. In dealing with animals, other sites may have to be chosen; for example, the best place in birds is the dorsum of the foot, in rats and mice the tail, in rabbits the ear, in big animals—horses and cows—also the ear. Whichever is selected, at least for the human subject, the part must be thoroughly cleaned and sterilised. Failure to do this might quite easily lead to septic or other troubles, and though this is excessively rare, still its occurrence would be very annoying both to the patient and the operator. The finger or ear, then, should first be cleaned with soap and water (the finger of a mechanic will require more vigorous scrubbing than that of a lady or clerk), then rubbed with 1-20 carbolic, then with alcohol and ether, then dried with a silk handkerchief to prevent fluff or pieces of cotton sticking to it. The part is then ready for puncture with an ordinary needle, a sharp lancet, or preferably with one of the small knives used in eye operations. The latter seems to give far less pain than the needle, and one can regulate the size of the wound much better, so as to get the necessary amount of blood (this may be considerable if a complete count is to be done). It is always advisable, especially in dealing with females, to get sufficient blood from the one puncture; many are very averse to have to be pricked again, and some even will absolutely refuse, so that the examination will only be partial and cannot be concluded. The first drop of blood that escapes should be wiped off (scales and other pieces of skin might be mingled with this), and the finger should not be unduly squeezed, the blood being encouraged to flow freely of its own accord. If squeezed too much serum would come out, and this dilution of the red cells would give fallacies in counting them with the hæmocytometer. If all that is required in the examination is to make fresh films only, then a small drop is enough, and one of three methods may be adopted for this purpose.

I. Catch up a small drop on a cover-glass, quickly drop this on a slide. If this spreads out uniformly between the two, it indicates a clean cover-glass and slide, and also a good film.

II. What is sometimes known as Braddon's method. Place a square cover-glass on a slide, and ring it all round with vaseline except one free edge and a small aperture opposite. Place this free edge in contact with drop, the blood flows in by capillary attraction and forms a very thin, regular film.

III. Make film between two cover-glasses (lower longer than top one); clamp these on slide.

Fresh Blood Films.

A well-made film should show three distinct zones: (1) an outside zone, red and irregular: here the corpuscles are heaped up in rouleaux or masses; (2) the opaque or ground-glass area: here the corpuscles lie out flat and do not overlap each other; (3) the central, clear, transparent zone: here there are only single corpuscles, few in number. The second or ground-glass zone is the best one for studying the individual corpuscles or for intra-corpuscular parasites; the outside zone is useful for extra-corpuscular parasites (trypanosomes, crescents, etc.), especially if they are scanty, and also for microscopically gross parasites, such as filaria. Films tend to dry up, especially when the weather is hot, so if the examination is to be prolonged, over fifteen or thirty minutes, say, one should ring the slide entirely round with vaseline to prevent evaporation.

There are many things to see and study in fresh films of normal blood. Normally the erythrocytes, or red blood corpuscles, are circular, biconcave, non-nucleated discs measuring 7.5 $\mu$ in diameter. One should specially note (1) their size, (2) their shape, (3) their colour, and (4) the presence of fallacies. Their size is, as just mentioned, 7.5 $\mu$ across, but in certain anæmias, as will afterwards be shown, much larger or much smaller diameters may be met with. Their shape, nominally circular, biconcave discs, may in some instances become altered to cocked-hat or sausage shapes; the term applied to this irregularity is poikilocytosis, and the individual irregular cell is termed a poikilocyte. Blood, of course, appears red in mass. Single corpuscles under the microscope are of a yellowish green shade. Practice enables one very quickly to detect differences in the colour of the blood in different diseases when it flows from the finger. In anæmias the colour is less red and of a much more watery consistence: in severe cases this being very marked. In leukæmia the blood is sticky and forms films on slides with greater difficulty than usual; if there is coincident anæmia of any degree, then it will be pale in colour as well as sticky.

Fallacies in Blood Films.

Fallacies and artefarcts are of special importance, because they are constantly being mistaken by the uninitiated for parasites. They do not exist normally in the blood within the body; they are produced artificially, either by contact with the air, or by extraneous matter getting blown on to the slide or being there when the films are made (dirty and faulty technique). The pressure also required in making the film must necessarily mechanically distort many of the cells, and such distortion must not be mistaken for poikilocytosis. The chief of these artificial conditions are crenation, cracks in the corpuscles, vacuolation, buckling of corpuscles, fibres of cotton, specks of dust, etc. The diagnosis of vacuoles, with a little practice, is easy; they are indefinite in outline, often stand out sharply, do not move, and should, of course, be non-pigmented. Their shape varies, a circular one in the centre of the corpuscle being one of the commonest. Of others, an eye-shaped one may be seen, and this often appears to have two little specks of pigment in it. Buckling of corpuscles, *i.e.* doubling of the cell on itself, often produces peculiar shapes, one of these resembling very closely a malarial crescent. It can, however, be at once told by the fact that there is no dark black pigment in its centre.

Varieties of White Corpuscles.

The different varieties of leucocytes can quite easily be seen in fresh blood, but as they are more conveniently studied in stained films, their description will be deferred, the names only being mentioned now. Five forms are usually described in normal blood, some of these having a single, others a polymorphous nucleus.

| | |
|---|---|
| I. Polymorphonuclear | 1. The common polymorphonuclear.<br>2. The eosinophile. |
| II. Uni or mononuclear | 3. The small lymphocyte.<br>4. The large mononuclear.<br>5. The transitional. |

Of these, 1 and 3 will easily be seen in any normal blood, 2, 4 and 5 less commonly, or even practically not at all. The eosinophile is very easily told on account of its large, highly refractile granules, and the shape of the nucleus and size of the cells will help in the differentiation of the others.

Blood-plates are very difficult to see in fresh blood. They are little, colourless, circular, or slightly oval-shaped bodies, measuring about 2 to 3 $\mu$. Normally they number 400,000 to 700,000 per c.mm., but as their estimation is a somewhat difficult task, they are very seldom counted in ordinary blood examinations. They are said to form the chief constituents of white thrombi, and are concerned in the processes of thrombosis; when diminished in number, clotting is slow—for example, in purpura and hæmophilia.

What has been termed Muller's blood dust is a condition often noted in ordinary fresh blood films. This so-called dust consists of small, round, colourless granules about $\frac{1}{4}$ to 1 $\mu$. in diameter, highly refractile, and with a rapid dancing movement. The minute bodies are insoluble in alcohol and ether, and take no part in the formation of fibrin. They are probably the extruded granules of eosinophile leucocytes, and are, of course, of no importance except in so far as they may be mistaken for cocci or other organisms. To see them at all, of course, a very high power of the microscope must be used—$\frac{1}{12}$-inch objective, with a No. 4 or other high eye-piece.

When a wet film is becoming old (this takes place more quickly if the film has not been ringed with vaseline), fine threads begin to appear among the corpuscles. These are threads of fibrin, and indicate the ordinary process of coagulation. If one examines a wet film of blood under the microscope and lightly touches the cover-slip, the corpuscles all move, showing that they are floating in an invisible medium. This medium is the serum of the blood, and, as it is of importance, its description may be delayed till next article.

